# Advancing bridge resilience: a review of monitoring technologies for flood-prone infrastructure

**DOI:** 10.12688/openreseurope.19232.1

**Published:** 2025-01-27

**Authors:** Karina Buka-Vaivade, Vanni Nicoletti, Fabrizio Gara

**Affiliations:** 1 DICEA—Department of Construction, Civil Engineering and Architecture, Università Politecnica delle Marche, Ancona, 60131, Italy

**Keywords:** Structural Health Monitoring, bridge failures, resilient infrastructure, scour monitoring, hydrodynamic monitoring, debris impact on bridges, flood risk mitigation, climate change

## Abstract

Floods pose a critical threat to bridge infrastructure, which plays an essential role in transportation networks and economic resilience. This review examines state-of-the-art Structural Health Monitoring (SHM) technologies tailored to mitigate flood risks, focusing on their real-world applications in flood-prone bridges. A central feature of this review is the extensive use of case studies, illustrating diverse SHM methods applied globally to monitor challenges such as debris accumulation, hydrodynamic forces, and scour—primary causes of bridge failures. These examples provide detailed insights into technologies like sonar-based devices, scour probes, photographic monitoring, rotation- and vibration-based techniques. By showcasing specific case studies—such as bridges monitored using smart magnetic rocks, Interferometric Synthetic Aperture Radar (InSAR), and fibre optic sensors—the review highlights practical outcomes, demonstrating how SHM systems enhance resilience through early detection and predictive maintenance. It also explores the challenges of implementing these systems, including environmental sensitivity, cost, and data complexity, while identifying gaps in integrating hydraulic and structural data for holistic risk assessments. This review advocates for multidisciplinary collaboration and advanced data-driven solutions, such as AI-based predictive maintenance, to address climate change impacts and increasing flood risks. By bridging cutting-edge research with real-world applications, this article provides actionable insights into scalable, adaptive SHM solutions, inspiring engineers and researchers to develop more resilient infrastructure for a changing world.

## Plain Language Summary

Bridges are vital for our daily lives, connecting communities, enabling trade, and supporting emergency responses. However, floods, which are becoming more frequent and severe due to climate change, pose a major threat to bridge safety. This review explores how new technologies help monitor and protect bridges from damage caused by floods. It explains why bridges fail during floods, such as when fast-moving water erodes their foundations, debris piles up against them, or powerful water forces overwhelm their structure. The review highlights real-world examples from around the globe, showing how sensors, cameras, and other tools are being used to detect early signs of danger. These technologies, like sonar systems that monitor underwater erosion or cameras that track debris, provide real-time information, allowing engineers to act before a disaster occurs. The article also discusses how combining different monitoring methods with advanced data analysis, including artificial intelligence, can improve bridge safety. By focusing on practical examples and innovative solutions, this review emphasizes the importance of preparing our infrastructure for a changing climate, ensuring that bridges remain safe and reliable for generations to come.

## 1. Introduction

Bridges are critical transportation infrastructure components, facilitating the movement of people, goods, and services, and ensuring economic stability, social connectivity, and effective disaster response. Their structural integrity is essential for maintaining these functions, as demonstrated by recent events. For instance, on September 11, 2024, the partial collapse of the Carola Bridge in Dresden, Germany, not only caused major traffic disruptions, halting both car and ship traffic but also led to a citywide hot water outage due to the bursting of two large district heating pipes. Fortunately, there were no victims, which would otherwise greatly increase the damage count.

Bridges worldwide are particularly vulnerable to hydraulic forces during flood events, which can lead to catastrophic damage or even collapse. In the United States, data from 1992 to 2017 show that approximately 55% of bridge collapses, totalling 239 bridges, were caused by hydraulic factors, including flooding, scour, and debris accumulation ^1– 4^. Among these, scour, or sediment erosion around bridge foundations by fast-moving water is the most frequent cause of hydraulic failures in shallow foundations ^5, 6^. The risk is not limited to the U.S.; for example, scour is recognised as the leading cause of bridge failure in the UK and Ireland, accounting for 140 railway bridge failures during 65 separate flood events between 1846 and 2013 ^7^. Projections indicate that due to climate change, 1 in 20 bridges in the United Kingdom will face a high risk of failure within the next 60 years ^8^. In China, data from 2007 to 2015 show that floods were responsible for 44 out of 102 bridge collapses, accounting for 43.1% of failures. Of these, 17 were arch bridges (38.6% of the collapsed bridges), and 18 were beam bridges (40.9% of the collapsed bridges) ^9^.

**Figure 1.**
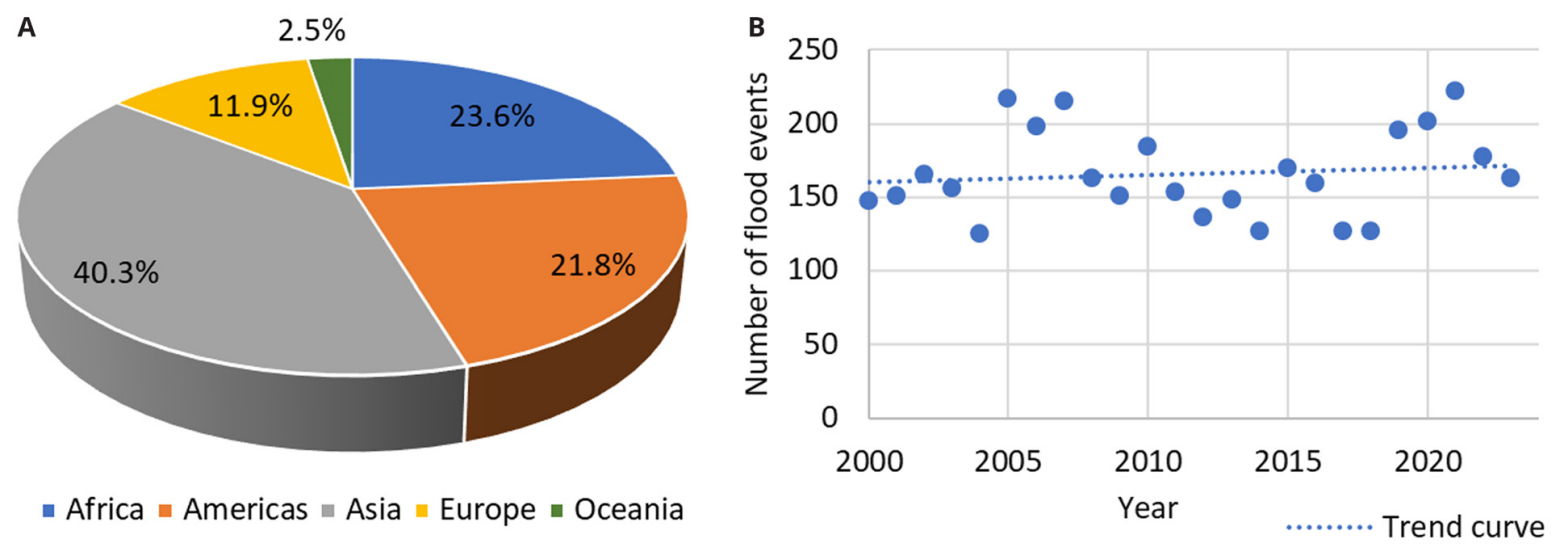
Worldwide flood events from 2000 to 2023, categorised by: **a**) geographic regions; **b**) annual distribution.

According to the EM-DAT database, which tracks global natural disasters, 3,977 flood events were recorded worldwide from 2000 to 2023. While not all of these events directly affected bridges, they caused significant damage and loss of life, with 130,379 fatalities recorded over this period ^10^. As shown in Figure 1b), flooding events display a cyclical pattern over the years; however, an upward trend is evident in the last two decades.

The Paris Agreement of 2015 aims to limit global warming to well below 2°C above pre-industrial levels, keeping the increase to 1.5°C. However, under current national policies, the world is on track for a 2.8°C warming pathway ^11^.

Studies show a strong correlation between atmospheric warming and increased flood risk on a global scale. At 4°C of global warming, countries representing over 70% of the global population and gross domestic product are projected to experience a flood risk increase of more than 500%. Even under the most optimistic scenario of 1.5°C warming, global flood risk is expected to more than double compared to the period from 1976–2005. These changes in flood risk are unevenly distributed, with the largest increases projected for Asia, the U.S., and Europe ^12, 13^.

As climate change leads to more frequent and intense flooding globally, bridges are increasingly at risk of damage or collapse due to hydraulic forces. Moreover, bridges in mountainous regions face particularly high structural failure risks, as steep topography amplifies water-flow intensity during flood events ^14^. This escalating threat underscores the need for comprehensive monitoring and assessment of bridge structural health. Effective Structural Health Monitoring (SHM) systems are essential for identifying vulnerabilities early, allowing for timely preventive measures to reduce the likelihood of catastrophic failure. By utilising advanced sensors to track changes in structural performance, SHM systems provide real-time data on bridge integrity, particularly during extreme flood events, enabling proactive responses that enhance safety and resilience ^15– 19^.

The role of SHM is especially important in flood-prone regions, where SHM systems can be a vital tool for protecting infrastructure, reducing maintenance costs, and, most importantly, saving lives ^20– 22^. Current research on bridge monitoring focuses on the challenges of data capture and interpretation.

This review aims to provide a comprehensive evaluation of SHM technologies applied to flood-prone bridges and related challenges, highlighting their strengths, limitations, and the potential for hybrid integration to enhance infrastructure resilience under extreme conditions. The main focus is on recent advancements in SHM technologies, specifically those published since 2018, to highlight contemporary solutions for monitoring flood-prone bridges. While this approach excludes older methods, it ensures an emphasis on state-of-the-art technologies and their applicability to current and future challenges.

Section 2 provides an overview of flood-related bridge failures and risks, highlighting real-world bridge failures due to flood events and potential preventive measures. Section 3 discusses recent advancements in monitoring technologies for assessing debris accumulation, hydrodynamic forces, and scour, illustrated with case studies. A summary and future outlook are presented in Section 4.

## 2. Flood-related bridge failures and risks

Floods pose significant bridge risks, causing immediate and long-term structural issues. The primary types of flood-related damage include ^23^:

- Debris impacts
- Hydrodynamic forces
- Scour and erosion

While some of these risks can be mitigated through preventive measures, structural monitoring is crucial for detecting and managing vulnerabilities, particularly during extreme flood events. Human factors also contribute to flood-related bridge collapses, including ^8, 24– 26^:

- Insufficient hydrological data for accurately estimating flood magnitudes during design.
- Lack of reliable methods for predicting scour at bridge piers.
- Inability to model and anticipate debris impact and accumulation during floods.

Moreover, inadequate maintenance and poor management practices exacerbate these risks, further heightening the vulnerability of bridges to hydraulic forces. Solutions such as flood-resilient designs, improved scour protection, and foundation reinforcements are crucial to ensure bridge stability during extreme weather events. The implementation of advanced hydrological models, debris control systems, and consistent maintenance protocols can further safeguard these critical infrastructures from failure during floods. Table 1 summarises a few recent flood-related bridge failures, highlighting the diverse impacts of flooding events on bridge infrastructure across different regions.

**Table 1.** Some recent bridge damages and collapses due to floods.

#	Bridge name, location	Date	Failure cause	Description
1	Misasa Railroad Bridge and 22 out of 94 nearby bridges, Japan ^39^	July 2018	Hydrodynamic forces, and scour	Flood-induced forces nearly doubled those used in seismic design ^40^. 11 bridges had collapsed, 6 had localised scour, 4 had abutment erosion, and 1 had other damage.
2	Baringin Bridge, Indonesia ^41^	November 2018	Debris impact	Large wooden debris damaged the temporary support structure, causing a collapse of the bridge during its construction.
3	Jinshajiang Bridge, China ^42^	November 2018	Hydrodynamic forces	Seven out of ten spans were washed away when the water rose 4 meters above the bridge deck.
4	Catalonia Bridges, Spain ^2^	October 2019	Debris accumulation	Flash floods clogged bridges with debris, causing collapses and flooding in nearby areas.
5	15 bridges in Thessaly, Greece ^14^	September 2020	Overflowing, debris accumulation, scour	Various damages following the Cyclone Ianos.
6	Fitzroy River Bridge, Australia	January 2023	Erosion, debris	Exceptional floods caused by rainfall equalled the past twenty years' worth in 24 hours. Floodwaters with debris washed away one of the piers, causing significant bridge damage.
7	Randklev Railway steel-truss Bridge, Norway	August 2023	Foundation failure caused by hydrodynamic forces	Despite the good condition of the bridge, approved by regular inspections, the foundation failed on the riverbed, sinking two bridge elements into the river. Traffic was suspended because of a storm warning, preventing injuries or fatalities.
8	Malo Bridge, Italy	May 2024	Debris impact	Small viaduct collapsed under debris load in floodwaters.
9	Visletto arch-type Bridge, Switzerland	June 2024	Hydrodynamic overload	River flow increased from 25 to 2,000 cubic meters per second, overwhelming the bridge.
10	Railway bridge, South Dacota, US	June 2024	Hydrodynamic forces	After heavy rainfall, floodwaters surged over the steel bridge, ultimately causing its collapse.
11	Shaanxi Bridge, China	July 2024	Scour	40-meter deck section of just 6-year-old bridge collapsed during flash floods with a maximum inflow of 1,640 cubic meters per second; 38 fatalities were reported.
12	Phong Chau Bridge, Vietnam ^43^	September 2024	Scour	Intermediate support scoured during floods; 13 people missing.
13	Kinser Bridge, Tennessee, US	September 2024	Hydrodynamic forces	The bridge was swept away after the water level rose by at least 18 meters.
14	Pedestrian Bridge in Carcare, Italy	October 2024	Debris accumulation	Logs formed a blockage, increasing hydrodynamic forces and leading to damage.
15	Paiporta Bridge, Spain	October 2024	Hydrodynamic forces	Bridge destroyed in seconds; rainfall equalled a year’s worth in eight hours.

These incidents underscore the urgent need to account for increasingly severe flood scenarios in future bridge designs and to continuously control the existing ones during floods to prevent such catastrophic failures.

### Debris impacts

Floodwaters carrying debris present serious risks to bridges, both by directly impacting the structure and by altering water flow around piers and abutments. Debris and mud flows are often characterised by high velocities, occasionally exceeding 10 m/s, and can have densities up to twice that of water ^27^. Debris carried by swift currents, such as trees, rocks, and even vehicles, can collide with bridge components, exerting significant impact forces that compromise the structural integrity of the piers. Smaller bridges are particularly susceptible to debris-induced damage (see Table 1, cases #8 and #14).

**Figure 2.**
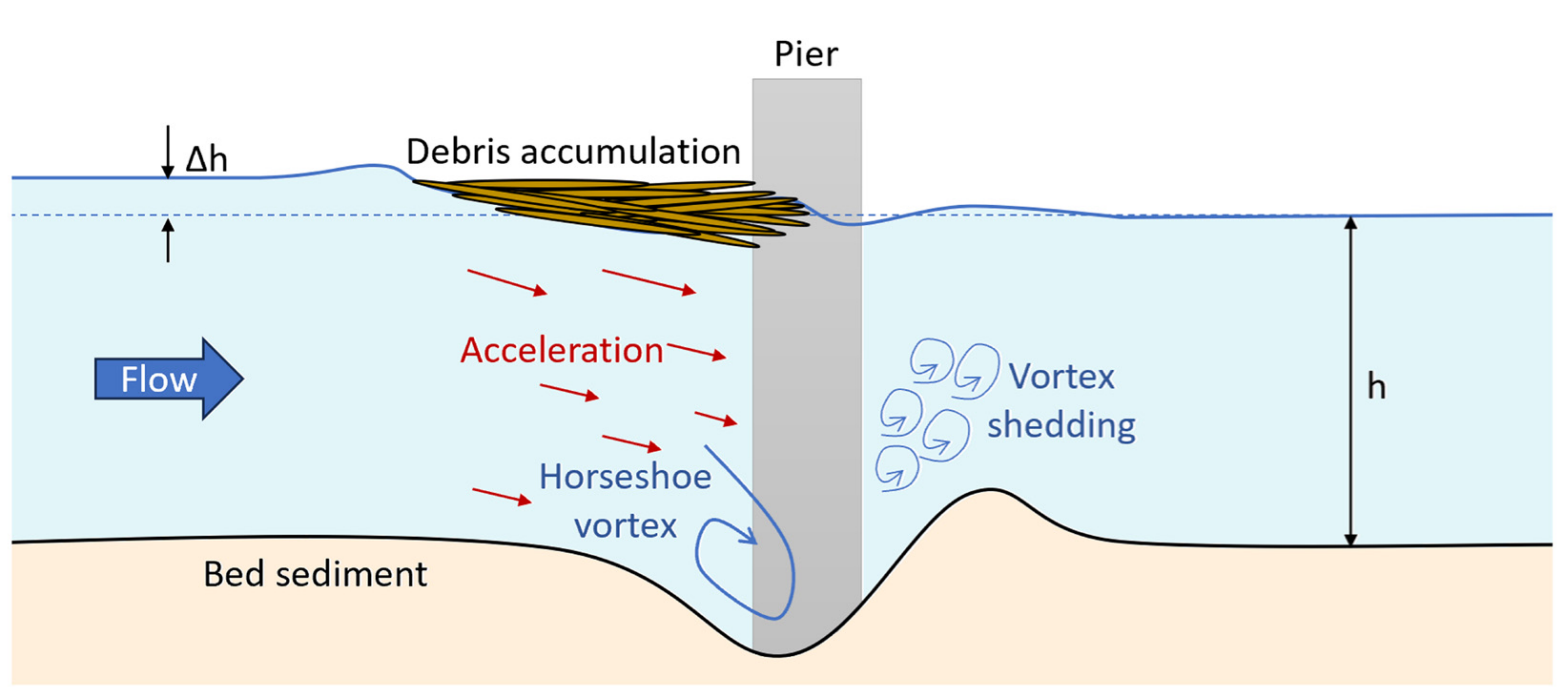
Scour around bridge piers exacerbated by debris accumulation.

Debris accumulation around piers significantly intensifies hydrodynamic forces ^1^, which can be estimated using simplified empirical models. These models typically use flow characteristics such as water depth, width, velocity, and the size of the debris pile to calculate a drag coefficient and, consequently, the drag force acting on the debris jam ^28^. This drag force on the debris pile translates into an additional load on the bridge pier, increasing the overall hydrodynamic force that the structure must withstand. Moreover, debris acts as a partial blockage, disrupting the flow and creating localised pressure variations. This blockage raises the upstream water level, increasing pressure on the bridge structure ^29, 30^. Simultaneously, as shown in Figure 2, the reduced flow area beneath the debris, driven by the principle of continuity, accelerates the water, increasing its erosive power and promoting local scour around the piers. This acceleration and altered flow patterns generate vortices, which further intensify the scouring effect at the bridge foundation ^31– 34^. The cumulative effect of debris accumulation, increased flow velocities, and intensified scour severely threaten bridge stability. If left unchecked, such conditions can lead to progressive structural damage, potentially resulting in partial or full bridge collapse, especially during extreme flood events ^32^.

**Figure 3.**
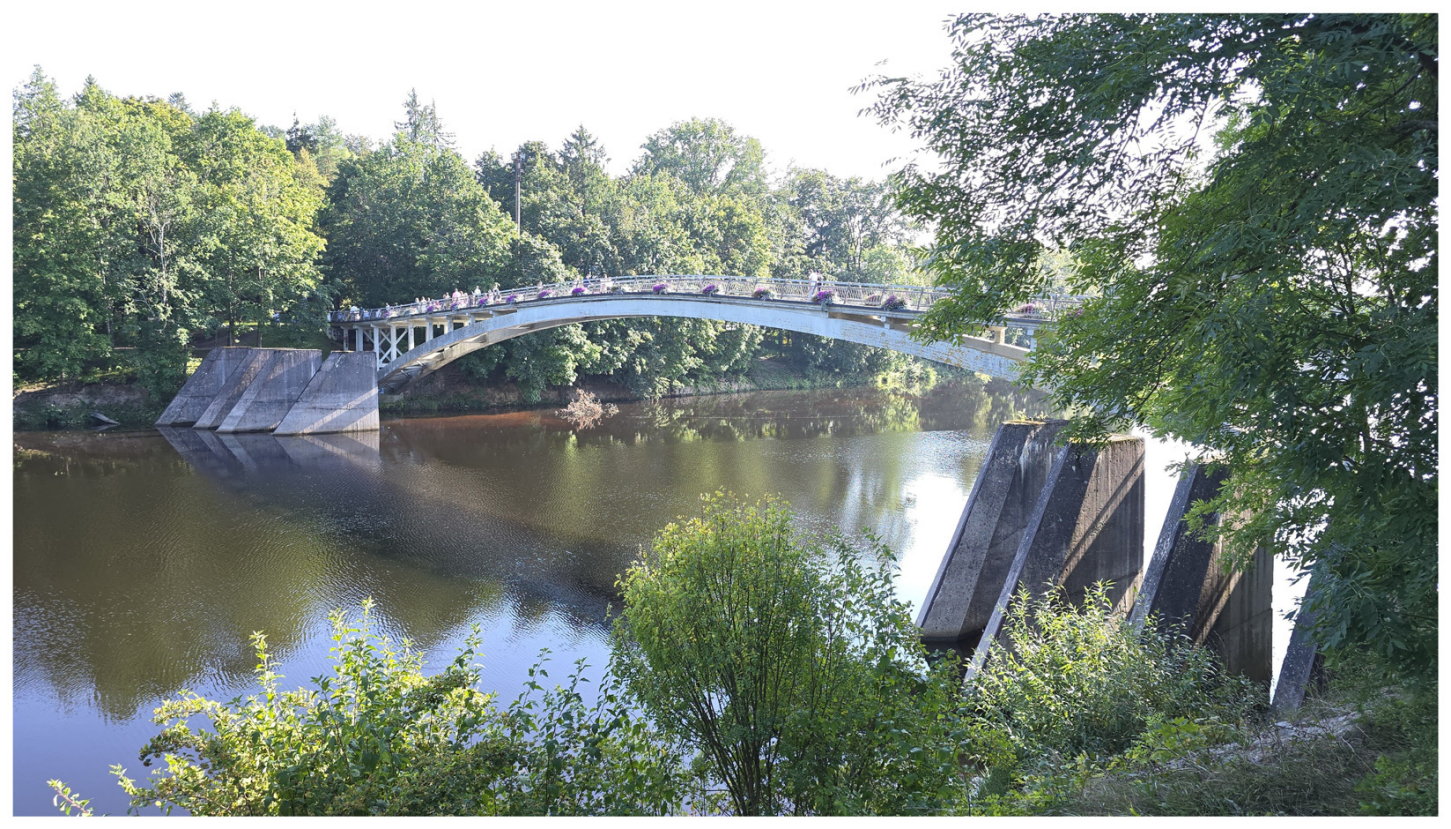
An example of protective measure for bridge piers, including barriers to shield against water flow and debris.

To mitigate these risks, debris deflectors or protective barriers can be installed in front of bridge foundations. These barriers ( Figure 3) deflect debris, reduce blockages, and prevent structural damage ^35, 36^. However, their design and placement must be carefully tailored to specific flood and debris flow conditions.

### Hydrodynamic forces

The force exerted by fast-moving floodwaters on bridges can cause structural deformations, displacements, and even collapse. This hydrodynamic failure is particularly significant for bridges with foundations such as piers with spread footings on bedrock, where scour might not be the primary failure mode ^37^.

Stream-crossing bridges are exposed to multiple hydrodynamic effects ^38^:

- Hydrostatic forces, caused by differences in water levels on both sides of submerged bridge components (e.g., piers, deck).
- Buoyant forces, equal to the weight of the water displaced by submerged bridge structures.
- Hydrodynamic forces, including drag and lift forces.
- Overturning moments, created by uneven forces applied to the bridge.

Hydrodynamic forces during floods consist of two primary components: drag force and lift force. Drag force acts parallel to the flow, pushing the bridge downstream, while lift force acts perpendicular to the flow, either pushing the structure upward (positive lift) or downward (negative lift). For bridge decks, particularly during submersion, lift forces can threaten to lift the deck off its supports, leading to failure.

The intensity of these forces is influenced by the velocity of the water, with a quadratic relationship between hydrodynamic forces and water flow velocity ^37, 44^. Additionally, hydrodynamic pressure is affected by water depth: as the depth increases, the volume of water interacting with the structure grows, increasing the pressure exerted on the bridge.

If the bridge deck becomes submerged during a flood, it faces increased horizontal forces and greater static water pressure. Submerged decks can act like dams, further increasing the load and potentially causing structural failure. Therefore, hydrodynamic deck failure can occur on all bridges, regardless of foundation type ^37^.

Accurately estimating flood-induced hydrodynamic loads, especially on bridge superstructures, is critical for designing flood-resilient bridges ^1, 45^. For instance, bridge design standards in various countries, such as the Eurocode (EN 1991-1-6), U.S. AASHTO, and Australian AS 5100 codes, account for similar types of forces ^38^.

### Scour and erosion

Scour is the process of erosion and removal of sediment from around bridge foundations, piers, or abutments caused by the increased velocity of water flow near the structure. It is the leading cause of failures for bridges with shallow foundations during floods, as it undermines the stability of a bridge by removing the supporting material, which weakens the foundations and can potentially lead to collapse. As scour progresses, it increases the unsupported length of the foundation, reducing its lateral stiffness, strength, and buckling resistance, while also lowering the factor of safety against structural instability ^46^.

Interestingly, both bridges recently damaged by scour ( Table 1, cases #11 and #12) featured round-shaped and oblong-shaped piers, respectively. Round-nose designs are generally considered the standard baseline for scour resistance.

**Figure 4.**
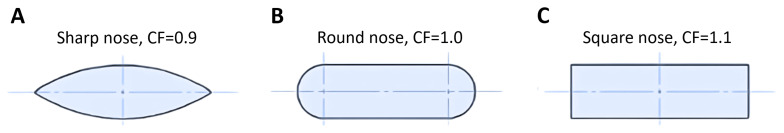
Correction factors (CF) for various pier shapes: **a**) lenticular, **b**) oblong, **c**) rectangular.

Research indicates that pier shape significantly impacts scour depth, with correction factors providing a measurable indicator of a shape's effectiveness against scour ^47– 49^. Square and rectangular shapes are the least effective, as they increase turbulence and vortex intensity, leading to more severe sediment removal around the foundation. In contrast, shapes with a sharp nose and curved body, such as lenticular piers, reduce turbulence, allowing for smoother water flow around the pier and minimising vortex formation and sediment removal. These designs are typically associated with enhanced scour resistance. Correction factors (CF) for various pier shapes are summarised in Figure 4 ^50, 51^.

Despite round-nose optimised designs, piers still experienced significant scour damage, underscoring that even with favourable geometry, bridges can remain vulnerable to extreme flood conditions. So, the use of scour protection systems and continuous monitoring are always recommended.

Scour is particularly dangerous because it often occurs below the waterline, making it difficult to detect without proper monitoring systems. A notable example of this occurred in November 2000 when a major flood, affected a state road bridge crossing the Po River near Borgoforte, Italy. Three months after the flood, a bathymetric survey revealed a 15-meter-deep scour hole near two central piers in the river’s main channel. It is believed that the scour depths could have been even greater during the flood event than what was recorded post-event ^52^.

Traditional deterministic methods provide qualitative indicators of scour risk by combining the likelihood and consequences of failure, but they do not offer precise measures of scour vulnerability ^53^. Numerous studies focus on the development of precise predictive models for scour depth estimation. For example, one recent study investigated a prediction method for the temporal evolution of local scour depth at bridge piers during floods, based on the law of conservation of energy ^54^. Another approach utilised Bayesian Networks to propagate information from scour probes and gauging stations, updating the probabilistic distribution of scour at unmonitored bridge foundations across a network ^55^.

High-importance bridges require regular visual inspections, especially after hazards ^55, 56^. However, the challenge with scour is that it is often only detectable after a flood, and sediment deposition during the event can obscure the extent of damage. This makes visual inspections prone to errors, complicating the accurate assessment of scour and increasing the potential for human error in interpretation ^8, 57^.

Similar to scour but more widespread, erosion involves the gradual loss of soil and sediment surrounding the bridge’s structural component foundations. Over time, this can weaken the support structures, leading to instability, misalignment, or collapse. Riverbanks and embankments near bridges are particularly susceptible to erosion during prolonged floods or high-flow events.

Mitigation measures such as pilings, riprap, revetment, gabion mattresses (rock or other material used to protect foundations from erosion) ^8, 58^, scour protection devices, and real-time monitoring systems are essential for identifying and managing these scour-associated risks. Furthermore, understanding local hydrological conditions and flood patterns is crucial for designing effective protective measures that can withstand the long-term effects of scour and erosion.

## 3. SHM technologies and methods for remote monitoring bridges during and after floods

SHM plays a vital role in minimising flood risks by providing remote monitoring, enabling early warnings, and supporting structural evaluations during and after floods. While preventive measures, such as barriers, help mitigate debris impact, SHM systems are invaluable for detecting unexpected stress or vibrations from collisions. These systems enable rapid assessments and timely interventions, such as temporarily closing the bridge to prevent further damage. Similarly, real-time monitoring of hydrodynamic forces during extreme weather events allows proactive responses to prevent failures. Due to scour long-term and progressive nature, advanced SHM systems are essential for detecting early signs of scour, the leading cause of flood-related bridge failures. Scour and erosion detection strategies can be classified as direct and indirect, depending on whether detection and measuring are performed directly on scour at a particular location or if they measure the effects of scour on the bridge ^59, 60^. Technologies like sonar, scour probes, float-out devices and smart rocks provide direct insights into sediment movement, while vibration-based sensors, tiltmeters, and Interferometric Synthetic Aperture Radar (InSAR) offer indirect clues by monitoring the structure's response and environmental conditions. While direct methods can detect the presence or progression of a scour hole, an additional SHM system is required to assess the overall condition of the bridge ^61^. The following sections discuss and summarise recent advancements in monitoring technologies, along with illustrative case studies.

### 3.1. Monitoring of debris accumulation by photographic imagery

Numerous studies investigated the effects of debris accumulation, taking into account the shape and position of the debris accumulation, on both final scour depth and horizontal loads acting on the pier ^62– 64^, evaluating these effects as significant. According to Eurocode (EN 1991-1-6, 2005) the impact of the debris (F _deb_) can be calculated by the formula ( 1):

$${\text{F}}_{\text{deb}}={\text{k}}_{\text{deb}}\times {\text{A}}_{\text{deb}}\times {\text{v}}_{\text{wa}}^{2},\;(1)$$

where k _deb_ is debris density parameter (kg/m ^2^), A _deb_ is area of obstruction presented by debris (m ^2^), and v _wa_ is mean speed of the water (m/s).

In the case of fully submerged abutment of single span arch bridge, floating debris impact loads on the superstructure can be more than 6 times higher than the combination of hydrostatic and hydrodynamic loads for the case of no debris ^65^.

The most common approach to monitoring debris accumulation upstream of piers is through photographic or video capture, providing a direct and effective method to assess the extent of debris buildup and its timing ^66^. This method was implemented in the monitoring of the multi-span brick masonry Candia Bridge over the Sesia River ^34^ and the Borgoforte Road Bridge over the Po River ^46^, both located in Italy, using video cameras. For monitoring the Aurence Bridge in France ^67^, a HIK Vision 4 MP WDR fixed network camera was used to observe the sensors. Following a flood event, the camera confirmed that a dead tree had twisted the sensor setup, which explained the abnormal data readings.

Image processing techniques, such as calculating the average grey level for the region of interest in each video frame, can further enhance debris monitoring capabilities ^68^. When paired with Artificial Intelligence (AI), cameras can alert operators to critical levels of debris accumulation that may require intervention. Additionally, high-speed cameras enable high-resolution image analysis, which can be used to measure the velocity of debris flow ^69^.

### 3.2. Hydrodynamic force monitoring

Hydrodynamic forces are traditionally considered during the design phase, but monitoring these forces can be essential during operation to provide timely alerts if water levels and forces approach or exceed design thresholds. Such monitoring enables informed decisions regarding bridge closure, evacuation, or urgent maintenance. According to Eurocode (EN 1991-1-6, 2005), the drag force (F _D_) on structures such as bridge piers can be calculated by the following formula:

$${\text{F}}_{\text{D}}=0.5\times \text{k}\times {\text{ρ}}_{\text{wa}}\times \text{h}\times \text{b}\times {\text{v}}_{\text{wa}}^{2},\;(2)$$

where k is shape factor, ρ _wa_ is density of water (kg/m ^3^), h is water depth (m), b is object width (m), and v _wa_ is mean speed of the water (m/s).

**Figure 5.**
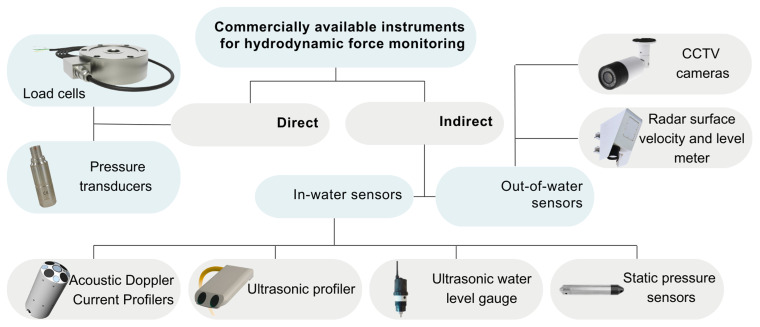
Overview of commercial instruments for hydrodynamic force and flow monitoring.

In practical monitoring applications, hydrodynamic drag forces can be measured directly using load cells and pressure sensors or inferred indirectly from water flow data from various other sensors ( Figure 5).

Direct measurements provide valuable data on flow-induced forces impacting the structure, though these sensors can be sensitive to structural vibrations and environmental conditions like temperature changes.

Several studies have explored methods to measure hydrodynamic pressure. For example, hydrodynamic forces on substructures have been evaluated in small-scale laboratory experiments and with computational fluid dynamics models ^38^. This study used the load cells and torque sensors, such as the Interface 3A100-100N-D11 load cell with a 100 N capacity and the Interface MRT2 torque cell with a 10 N-m capacity, to capture force and moment loads in three directions. A customised mounting frame was created to house both the load and torque cells, isolating the instruments from external vibrations and positioning them well above the water surface to prevent submersion-related damage. The load cells were connected to the submerged deck via vertical supports, which transfer the load from the submerged element. Notably, the load cell is also suitable for submerged applications when enclosed in a waterproof housing, as specified by the manufacturer.

For direct measurements of hydrodynamic pressure, pressure sensors or transducers are typically mounted on structural surfaces and are often manufactured specifically for submersion applications. Numerous laboratory studies have examined wave-structure interactions using pressure sensors for measuring wave loads. Examples include studies on wave force impacts on rectangular structures using standard pressure sensors ^70^, hydrodynamic forces from solitary waves interacting with submerged square barriers using pressure transducers ^71^, and pressure distributions caused by green water phenomena on structures, measured with Kistler 4043A2 piezo-resistive pressure sensors, which capture static and dynamic pressures within a range of 0 to 2 bars ^72^.

Pressure transducers, however, provide only single-point measurements, making it difficult to capture a comprehensive pressure field. As a result, multiple sensors are typically deployed to create a pressure map across the structure’s surface. These sensors also require careful calibration to ensure accuracy, which can be complex and time-consuming.

Fibre optic sensing is a rapidly growing field, offering advantages over traditional electrical strain gauges, such as high sensitivity, lightweight design, immunity to electromagnetic interference, long-distance detection, and ease of installation ^73– 75^. A common type, fibre Bragg gratings (FBGs), are widely used in civil engineering for strain and temperature monitoring. FBGs contain microstructures that reflect specific wavelengths of light, known as Bragg wavelengths, which shift when the fibre experiences strain or temperature changes. This shift allows precise monitoring of structural changes ^73, 76^. FBGs can also measure parameters like tilt, pressure, displacement, and vibration, though their high cost and limited end-user familiarity are drawbacks ^74, 77^. However, studies suggest FBGs could be a cost-effective solution for monitoring small- to medium-span bridges due to their reliability and low maintenance needs ^74, 75^.

Some studies have demonstrated FBGs for flow monitoring, using deformations caused by hydrodynamic pressures in single- and two-phase flows, as well as to measure substructure cross-sectional load for a semi-submersible floating wind turbine ^78, 79^.

Both load cells and FBG sensors require robust protective housing and secure mounting to withstand harsh environmental conditions and protect against potential debris impact.

There remains a gap in the literature concerning real-life applications of direct hydrodynamic force measurements in bridge monitoring systems.

For flow data, including water depth and velocity, acoustic-based instruments, especially Acoustic Doppler Current Profilers (ADCPs), are widely used. ADCPs, which emit sonar pulses, measure the speed and direction of water flow at different depths, offering precise flow velocity and water level data. However, ADCPs are costly, and acoustic instruments used for velocity measurement are vulnerable to debris damage. Their performance may also be limited in high-turbidity or flood conditions, as shown in studies monitoring the Rucúe Bridge in Chile with ADCPs ^80^ and the Aurence Bridge in France, where ultrasonic water level gauges and profilers were used ^67^.

Ultrasonic water level gauges measure water level by emitting sound waves and calculating the time taken for these waves to travel to the water surface and back. Since the speed of sound is affected by air temperature, temperature variations can cause measurement errors. So, temperature compensation is necessary for ultrasonic water level sensors ^81^.

There is another type of pressure sensor, designed for static or slowly varying pressure environments, primarily used for water level and depth measurements. These sensors are employed in hydrodynamic monitoring by submerging them at a specific point in the water column to measure absolute pressure, which is the sum of water pressure and atmospheric pressure. The pressure readings are converted to water depth, as illustrated in the Rucúe Bridge monitoring project ^80^. However, the accuracy of pressure sensors can be affected by factors like water temperature, salinity, and sediment content, making them more suitable for controlled environments.

For non-contact water level monitoring, instruments installed above the water surface, such as radar level sensors and closed-circuit television (CCTV) cameras, provide alternatives with fewer environmental vulnerabilities. Radar level sensors emit microwave pulses that reflect off the water surface, with the time delay of the return pulse used to calculate the water level. This method has proven effective in bridge monitoring studies, such as at the A71 motorway viaduct over the Loire River in Orléans, France ^82^.

Considering the vulnerability of the in-water sensors, and that the mean flow velocity can be estimated from the surface velocity from an out-of-water sensor, which is a more robust solution in a flood context, the non-contact sensors for flow surface velocity can be of interest. For example, a hydrologic station (RSS-2-300 WL) installed on the bridge over Aggitis River, near Simvoli Dam in Greece, has an integrated radar surface velocity and level meter for contactless measurements of surface flow velocity and water level at the current location ^83^.

Similarly, CCTV cameras are increasingly used in real-time floodwater monitoring, providing visual assessments of water levels and flow patterns. For instance, the Da-Chia Bridge in Taiwan employs real-time CCTV imaging in combination with the Mask R-CNN deep learning model to monitor changes in water levels around bridge structures during floods ^84^. In another application, monitoring the hydrodynamic behaviour of the Tara channel near Taranto, Italy, a camera was installed to capture water flow at the bridge base. Using particle image velocimetry (PIV), this setup enabled accurate detection of flow patterns and velocities ^85^.

From the non-intrusive PIV data, velocity fields can be used to compute the pressure field by solving the Poisson pressure equation derived from the Navier-Stokes equations. This PIV-based pressure measurement method, validated in controlled studies on wave force measurements on rectangular structures ^70^, offers a promising approach for calculating hydrodynamic forces acting on bridge structures.

When available, flow data from nearby hydrometric stations offer a reliable reference point, correlating well with on-site sensor data. As seen in the A71 motorway viaduct monitoring study, nearby stations provide useful context, especially for long-term monitoring or during periods when on-site sensor readings may be less accurate ^82^.

Table 2 presents a comprehensive comparison of various indirect water flow monitoring techniques applied in real-world cases, highlighting their underlying principles, key parameters measured, deployment locations, advantages, limitations, and documented applications in case studies.

**Table 2.** Comparison of indirect water flow data monitoring techniques on real bridges.

Technology	Principle	Key parameters measured	Location	Pros	Cons	Application in real case studies
**ADCP**	Doppler effect (acoustic waves)	Flow velocity and direction, water level	In-water	High-precision, multi-depth profiling	High cost Vulnerable to debris Limited in flood conditions	Rucúe Bridge, Chile ^80^; A71 Viaduct, France ^82^
**Ultrasonic profiler (e.g. Ub-flow F156)**	Ultrasonic waves	Flow velocity profiles, water depth	In-water	Detailed profiling at multiple depths	Sensitive to air bubbles, debris interference	Aurence Bridge, France ^67^
**Ultrasonic water level gauge (e.g. LNU06V3-82-3G)**	Ultrasonic waves (echo measurement)	Water level	In-water	Non-contact, reliable in various conditions	Requires temperature compensation	Aurence Bridge, France ^67^
**Pressure sensors (e.g. HOBO U20)**	Hydrostatic pressure	Water depth	In-water	Affordable, compact	Affected by temperature, salinity, sediment	Rucúe Bridge, Chile ^80^
**Radar surface velocity and level meter (e.g. RSS-2-300 WL, Valeport VRS-20)**	Microwave reflection	Flow velocity, water level	Out-of-water	Non-contact, reliable in harsh conditions	Limited to surface-level data at the current location	Simvoli Dam, Greece ^83^; A71 Viaduct, France ^82^
**CCTV cameras**	Visual imaging	Water level, debris presence, flow velocity	Out-of-water	Real-time flood tracking, cost-effective	Limited in poor visibility	Da-Chia Bridge, Taiwan ^84^; Candia Bridge over the Sesia River, Italy, ^34^; Aurence Bridge, France ^67^, Bridge over Tara channel near Taranto, Italy, ^85^
**Hydrometric measuring stations**	Hydraulic measurements	Flow velocity, water level	-	Reliable for regional flow data	May be located far from the site	A71 Viaduct, France ^82^; Kupa Karlovac Bridge, Croatia ^62^

### 3.3. Monitoring of scour and erosion

Most formulas for estimating scour depth are empirical, reflecting the complexity of interactions between flowing water, sediment properties, and structural elements. These interactions depend on various factors, including sediment type (cohesive or non-cohesive), flow conditions (e.g., clear water or live bed), and the geometry of the structure (e.g., pier or abutment shape) ^67^. Over 25 different methods for predicting equilibrium scour depth are summarised in the Appendix of 86.

**Figure 6.**
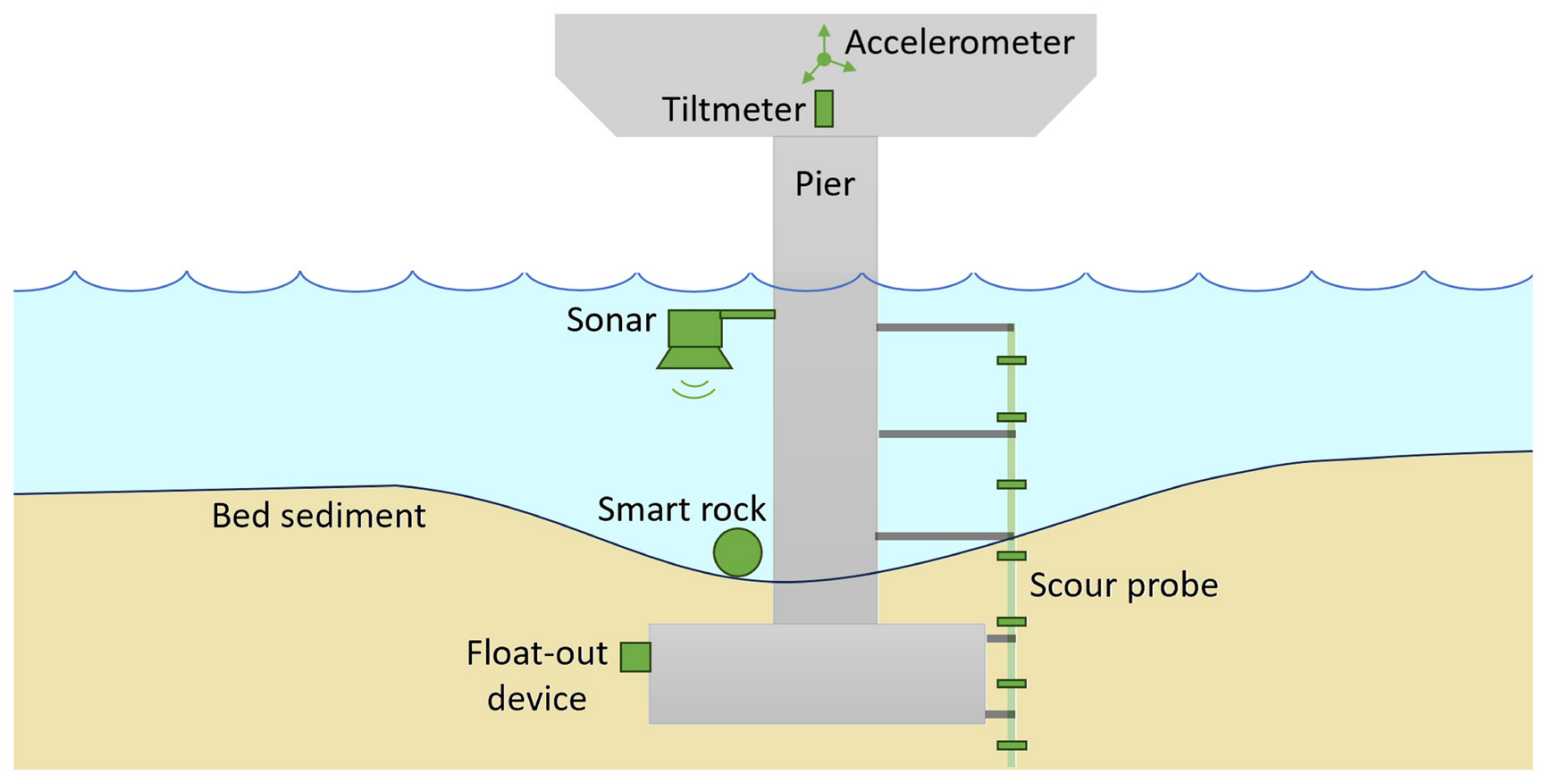
Instrumentation for monitoring scour and structural responses at bridge piers.

The primary limitations of scour depth formulas lie in their reliance on simplifications and limited datasets, reducing their accuracy and applicability to real-world conditions. Consequently, direct measurements of scour depth remain the most reliable approach. Figure 6 schematically illustrates the various instruments used to monitor scour and the associated structural responses, which are discussed in greater detail in the following sections.

In addition to scour depth direct measurements, emerging probabilistic frameworks for scour hazard assessment are gaining attention. These frameworks use data from monitoring systems to update the probability distribution of scour depth at the foundations of bridge networks ^55^.

To improve monitoring, it is recommended to measure water depth upstream of bridges to identify areas of greatest scour, typically occurring near the noses of bridge piers ^87^. By integrating direct measurements with advanced probabilistic modelling, a more robust strategy can be developed to manage scour risks and improve the resilience of hydraulic structures.

**i. Sonar or echo-sounding systems**

Sonar or echo-sounding systems use sound waves to monitor water depth and detect changes in the riverbed around bridge foundations. These systems emit sound waves that reflect off the riverbed or sediment, with the return time of these waves used to calculate distance. As erosion, or scour, occurs around bridge piers, the increasing distance to the riverbed indicates sediment loss. By capturing real-time bathymetric data, sonar systems provide critical insights into sediment movement below the waterline. Sonar-based instruments used to monitor underwater characteristics are commonly categorised into four main types:

- ADCP: is typically grouped separately from traditional sonar instruments, as it measures water velocity profiles, depth, and flow dynamics at multiple points in the water column rather than focusing solely on bottom mapping.
- Single-beam sonar: it emits a single sound beam, providing depth information along a line by measuring only at a single point in the water column. Though this limited measurement area can be seen as a drawback, it remains widely used as part of more complex monitoring setups. For example, a single-beam echosounder has monitored scour around a multi-span masonry arch, the Candia Bridge over the Sesia River in Italy ^34^. Pier-mounted sonars are also used in the permanent monitoring of over 15 bridges in Alaska, USA, by the United States Geological Survey (USGS), where real-time data is openly accessible ^87^. Another example is the Airmar Echorange SS510 ultrasonic scour sensor, which was mounted at a 9° angle on a rectangular pier of the Rucúe Bridge in Chile for scour monitoring ^80^.
- Multi-beam sonar: it emits multiple sound beams in a fan shape, covering a broader area to create detailed, high-resolution riverbed maps. This approach is valuable for long-term monitoring, enabling comprehensive coverage and high-resolution representation of scour ^60^. For instance, the Kongsberg Dual Axis SONAR was used to monitor sediment around the A71 motorway viaduct over the Loire River in Orléans, France ^82^. In another case, a Dual-Axis Scanning Sonar System installed upstream and downstream of a bridge pier provided full coverage of the Hangzhou Bay Sea Crossing Cable-Stayed Bridge in China, capturing accurate scour development data ^88^.
- Side-scan sonar: it uses sensors directed at an angle toward the riverbed to create detailed images of the underwater environment. Typically towed along the river or attached to a boat ^89^, side-scan sonar is well-suited for rapid underwater inspections ^90, 91^, though it may be less ideal for fixed installations at bridge piers ^92^.

Sonar-based systems, while powerful for real-time scour detection by plotting cross-sectional data, which can be used for comparison of obtained depth of the scour with allowed levels, share some common limitations. They operate only when submerged, making their effectiveness dependent on the water level of the river, which can lead to gaps in data during low-flow conditions ^34^. Additionally, they are sensitive to air bubbles, suspended sediments, turbulence, and debris interference, all of which can create gaps in data or false echoes and require careful filtering and processing ^34, 87, 88, 91, 93^. Table 3 compares different types of sonar systems, highlighting each one’s unique strengths, weaknesses and applications in monitoring scour.

**Table 3.** Comparison of sonar-based systems for scour monitoring.

Sonar system	Measurement method	Key parameters measured	Pros	Cons	Example use cases
**ADCP**	Doppler effect, multiple depths	Flow velocity and direction, water depth	Provides flow dynamics, multi-depth data	Costly	Rucúe Bridge, Chile ^80^
**Single-beam sonar**	Single sound beam	Depth at single points	Cost-effective, simple integration	Limited coverage, single-point data only	Candia Bridge, Italy ^34^; Alaska bridges ^77^
**Multi-beam sonar**	Multiple beams in fan shape	Detailed bathymetry	High-resolution, broad area coverage	Expensive, requires a complex setup	A71 Viaduct, France ^82^; Hangzhou Bay Bridge, China ^88^
**Side-scan sonar**	Angled sonar from moving vessel	Underwater imagery	Detailed mapping, ideal for inspections	Towed setup, limited to fixed locations	Rapid site inspections ^90, 91^

**ii. Scour probes**

Scour probes are a widely used technology for direct scour monitoring and have become a significant focus in recent research and field applications. These measure the position of the bed–flow interface near submerged structures usually by physical insertion into the riverbed. However, the insertion of probes may induce localised scour depths, potentially up to 2.5 times the probe's diameter ^94^. Scour probes are effective in environments where direct sediment erosion measurement is critical, offering both resilience and precision in harsh conditions.

Various types of scour probes, each with distinct measurement principles, have been successfully tested in real case studies or laboratory settings. Table 4 summarises notable examples of scour probes, highlighting their measurement principles, benefits, and specific use cases.

**Table 4.** Summary of scour probes used in scour monitoring studies.

Scour probe	Measurement principle	Pros	Cons	Example use cases
**EnviroSCAN probe with electromagnetic sensors along plastic rod height, encased in a protective steel sleeve, 10 cm resolution**	Electromagnetic sensing based on capacitance measurements. The sensor’s LC (L = Inductor, C = Capacitor) circuit resonance frequency changes based on the dielectric permittivity of the surrounding medium, which varies with sediment presence and erosion.	Stable during floods, accurate even in debris-prone conditions	Limited battery lifespan; the main board’s 16 channels reduce resolution at higher water levels	3-span stone masonry arch A76 200 Bridge over River Nith, Scotland ^94^,
**Micro-electro-mechanical systems (MEMS) sensors, packaged in a waterproof stainless-steel ball within a rebar cage, spaced at 50 cm intervals**	Vibration-based based on MEMS activation at multiple levels	Effectively capture scour and deposition processes during flood event	Continuous exposure to harsh environments demands regular maintenance	Da-Chia Bridge, 1.2 km long, Taiwan ^84^,
**Bed LEvel Seeking System (BLESS), patented by the Politecnico di Milano, with many Fibre Bragg Gratings placed from the bridge deck to several meters below the riverbed, spaced at 50 cm intervals**	FBG thermal sensing based on heat dispersion differences between water (convection) and ground (conduction)	Robust data, especially during extreme events; is useful as a cross-check when echo sounders fail	Placement downstream to avoid debris impacts, but major scouring often occurs upstream; may need heating for sensitivity; better calibration and choice for resolution are still under investigation.	Borgoforte bridge over river Po, Italy, 630 m long with four of the 44 permanently submerged piers ^52, 93, 95^
**Distributed fibre optic sensors with ultra-weak fibre Bragg gratings (UWFBG), installed in a spiral form on piers and embedded in soil**	The optical fibre detects deformation caused by water flow. Wavelength changes in the grating indicate the transition from soil to water, enabling scour depth estimation. The standard deviation of the wavelength signal is used as the scour indicator.	Real-time, high spatial resolution (5 cm). Flow velocity and temperature changes have minimal impact on the method's accuracy.	Turbulent water flow can introduce random variations in the wavelength, potentially affecting signal interpretation. Susceptible to failure if the optical fibre is damaged by debris; installation is limited to new bridges or during retrofits.	Laboratory test ^96^,
**Rod with an accelerometer at the middle**	Physical detection via cantilever model	Quick scour depth estimation, easier implementation	Rod material flexural rigidity must be much lower than soil stiffness; the water effect grows with scour increase	Laboratory testing ^97^,
**Frequency-based scour sensors with piezoelectric polymer strips**	Vibrational frequency analysis	The passive system operates during extreme events even if partially damaged	Requires complex calculations to account for fluid flow effects in the scour depths back-calculate from the obtained fundamental frequencies of the sensor.	Laboratory test ^98^,

**iii. Embedded sensors and smart rocks**

Using embedded sensors for direct scour monitoring is a practical, adaptable approach that has seen increasing applications. A basic form of these sensors includes passive "float-out" devices, which are simple yet effective fail-safe indicators for critical scour conditions. These devices are installed in high-risk scour zones near bridge foundations and operate by floating to the surface when the sediment erodes to their depth. This motion triggers an internal transmitter, which sends an alert to a data logger when the device surfaces, providing a direct indication of significant sediment loss ^95, 96^. Although they lack continuous data monitoring, float-out devices are valued for their straightforward operation and reliability in marking critical scour events. For continuous monitoring, smart rocks offer real-time insights into sediment behaviour and scour progression. Designed to resemble natural rocks, these engineered sensors are embedded in sediment around bridge piers and move with it as it erodes. Smart magnetic rocks are a commonly used variant, where erosion and deposition patterns are tracked by measuring magnetic fields using magnetometers ^95^. By mapping movement and positioning, these rocks provide valuable real-time data on sediment displacement and scour depth.

A notable validation of smart magnetic rocks was conducted on the Masangxi Bridge, a reinforced concrete cable-stayed structure over the Yangtze River in China ^97^. Here, smart rocks equipped with a neodymium permanent magnet and a three-gimbal gyroscope provided reliable scour depth data in flood conditions. However, environmental magnetic fields, such as those from the bridge and passing vehicles, introduced potential interference. Additionally, sediment redeposition can bury the smart rocks after a flood, necessitating careful placement and periodic maintenance to ensure reliable data.

To mitigate the impact of environmental magnetic fields, the use of two smart rocks has been suggested. Field tests conducted at the State Highway 1 Bridge (No. 36–0065) over Waddell Creek, Santa Cruz, California, USA, demonstrated that changes in the dispersion of ambient magnetic fields are more pronounced with double smart rocks compared to single ones. However, the effectiveness of the double smart rocks depends significantly on their relative locations, which influence the monitoring range ^102^.

Another example of the smart rock method was tested at the I-44 W Roubidoux Creek Bridge in Waynesville, USA ^103^, where an unmanned aerial vehicle (UAV)-deployed smart rock demonstrated precise measurements, with only a 6% error margin compared to sonar scanning. This smart rock consisted of a fibre-reinforced concrete shell with an inner and outer glass ball, filled with low-viscosity propylene glycol, containing two stacked N42 magnets and copper beads for balance. The rock’s design, including a vertical polarisation alignment, allows it to track flow velocity and water depth effectively. However, UAV deployment requires additional calculations for location-specific latitude and longitude, as well as assessments of nearby ferromagnetic materials.

**iv. Indirect monitoring methods: rotation and vibration-based monitoring, and InSAR**

Indirect monitoring methods, such as rotation-based and vibration-based monitoring, and InSAR, focus on the structural response of a bridge rather than directly measuring scour or sediment movement. These methods are particularly effective for assessing stability and load-bearing capacity when sediment erosion, scour, or hydrodynamic forces compromise structural integrity. Indirect monitoring approaches, commonly used for offshore wind turbine monopiles vulnerable to scour, share similarities with bridge pier monitoring. Critical parameters like bottom inclination, vibrational modes and natural frequency have been identified for scour detection ^104– 106^. These methods could inform bridge monitoring under similar conditions.

Extensive numerical analysis using a finite element model, calibrated by operational modal analysis (OMA) of the remaining part of the arch-type Rubbianello Bridge over the Aso River in Italy ^107, 108^, which was partly collapsed in 2013, recommended monitoring upstream rotations in piers or spandrel walls. For a multi-span brick masonry arch Candia Bridge over the Sesia River in Italy, a tiltmeter-based monitoring system was implemented to detect and localise structural damage from scour ^39^. Here, 15 uniaxial MEMS tiltmeters (SISGEO model 0S541MA0202, accuracy ±0.008°) with integrated temperature sensors were installed at the arch skewbacks on the upstream side to measure transverse rotations in the foundation-pier-arch system, thus protecting the sensors from debris. This setup allowed the identification of both local and global rotations, revealing an almost linear correlation between global rotations and temperature changes. Temperature-induced variations were filtered out to isolate abnormal rotational patterns, which could indicate structural issues. Tiltmeters proved effective in capturing abnormal behaviour during a minor earthquake and identifying anomalous patterns in global rotations. However, the study highlighted challenges in interpreting the data and suggested deploying additional tiltmeters on the downstream side to better distinguish between anomalies caused by arches and those from pier-foundation shifts.

Tiltmeters are primarily suited for quasi-static or slow-changing movements, so a new approach in rotation-based monitoring was proposed in 7, where a fusion of accelerometers and gyroscopes was used to achieve a more accurate and reliable estimate of structural rotation and deflection.

Scour affects the modal parameters of a bridge, making vibration-based sensors, typically employing accelerometers, valuable for monitoring scour ^53, 109^. These methods rely on modal analysis to detect scour-related changes in dynamic behaviour, such as shifts in natural frequencies, mode shapes, and mode-shape curvatures, rather than measuring scour depth directly ^110^. Natural frequencies generally decrease with structural damage ^111^, and numerical modelling of scour has shown increases in periods of the first two bridge modes and localised variations in mode shapes around scoured piers ^53^. However, for shallow foundations, natural frequencies are less sensitive to scour and predicted frequency variations from finite element models are often not observed in field testing ^112, 113^.

An advantage of monitoring mode shapes is their relative immunity to environmental and operational fluctuations, making them more reliable damage indicators than natural frequencies ^114^. For instance, the Rubbianello Bridge study demonstrated that transverse mode shapes were highly sensitive to localised scour at a single pier, even in the early stages of erosion, but this sensitivity decreased when multiple piers were affected ^59^.

In the monitoring of the A71 motorway viaduct over the Loire River in Orléans, France, accelerometers were deployed, including one tri-axial PCB 629A11 sensor under the bridge deck and two mono-axial PCB 393B31 sensors on the piers along the vertical and hydraulic flow axes. However, this study revealed challenges in post-processing vibration data and found no clear correlation between natural frequencies and water levels ^82^.

In the vibration-based monitoring of Baildon Bridge in Bradford, UK, during its scour repair program, alternative structural response parameters—spectral density and mode shapes—were proposed for capturing behavioural changes resulting from scour ^112^. Similarly, in the monitoring of the Hangzhou Bay Bridge in China, the variation of mode shapes was incorporated to qualitatively detect the presence of foundation scour ^115^. Comprehensive numerical modelling of a cable-stayed bridge tested four scour indicators: frequency change ratio, modal assurance criterion, mode shape curvature, and flexibility-based deflection. The study concluded that while the frequency change ratio can qualitatively identify scour, monitoring changes in flexibility-based deflection enables both qualitative and quantitative identification of scour in cable-stayed bridges ^116^.

Another notable example is the Z24 Bridge in Switzerland, which was monitored for nearly a year before scour was simulated by artificially lowering a pier. This study analysed the probability distribution function of natural frequency changes, with effective damage detection achieved through the use of quality control charts, specifically Hotelling’s T² statistic, implemented with P3P software ^117, 118^.

Introduced in 1947, Hotelling’s T² statistic is a multivariate control chart that combines information from the mean and dispersion of multiple interrelated variables to detect shifts in their collective behaviour. Although its calculations involve complex matrix algebra, modern software simplifies its application. This highlights the importance of advanced analytical tools in vibration-based scour monitoring for detecting subtle changes that simpler methods might overlook.

Another example of vibration-based scour monitoring is the application of time-frequency analysis combined with probabilistic trend change detection. This method, demonstrated in studies on the Jintang Bay Bridge and the Anqing Yangtze River Bridge (where scour frequently occurs) in China, leverages natural frequency data extracted from long-term SHM systems using techniques such as the Hilbert-Huang Transform and Short-Time Fourier Transform. Kernel Density Estimation is used to model the probability distribution of frequencies, which are normalised, and then control charts are used for anomaly detection. Continuous frequency deviations beyond preset thresholds effectively signal scour development, showcasing the method's ability to detect scour without underwater operations and its potential for integration with routine SHM systems ^119^.

Interferometric Synthetic Aperture Radar (InSAR) is a remote sensing technology that detects ground deformation by analysing phase differences between radar images captured from satellites. The temporal resolution of InSAR methods can be increased providing near-real-time monitoring possibilities by combining data from overlapping orbits ^120^. Advanced InSAR techniques, such as Persistent Scatterer Interferometry (PSI) and Small Baseline Subset (SBAS), allow accurate deformation monitoring over large spatial areas, making it particularly suitable for bridges under flood conditions or when direct monitoring is infeasible.

A notable application of InSAR was its deployment on the Tadcaster Bridge in the UK, where it detected deformation weeks before the bridge’s partial collapse due to scour ^121^. This highlighted its potential as an early warning system, complementing traditional visual inspections. However, interpreting InSAR data requires accounting for factors like expected thermal responses, bridge orientation, and water level variations, as seen in studies of the Jacques Cartier and Victoria Bridges in Montreal, Canada, and the A22 Po River Bridge in Italy ^122, 123^.

One key challenge of InSAR is that measured displacements represent one-dimensional projections along the satellite’s Line-of-Sight (LOS), which may not fully capture three-dimensional deformation patterns. Converting LOS data into a local reference system (longitudinal, transverse, and vertical components) is essential for understanding bridge behaviour. While longitudinal and vertical deformations are typically influenced by temperature effects and traffic loads, transverse deformations can become significant during events like landslides or localised scour around piers ^124^.

Table 5 summarises examples of indirect scour monitoring methods, highlighting their measurement principles, benefits, and specific use cases.

**Table 5.** Summary of indirect monitoring methods used in scour monitoring studies.

Method	Instruments	Measurement principle	Pros	Cons	Example use cases
**Rotation-based**	MEMS tiltmeters	Measures slight angular changes in bridge components due to foundation settlement or undercutting.	Effective for identifying foundation-pier-arch rotations. Easy to filter temperature variations. Protects sensors from debris through careful placement.	Data interpretation requires expertise. Distinguishing anomaly sources (e.g., arch movement vs. pier shifts) is challenging without additional sensors.	Candia Bridge over the Sesia River, Italy ^34^
**Rotation-based**	High-performance MEMS gyroscopes, high-sensitivity MEMS triaxial and ultra-sensitive piezoelectric quartz uniaxial accelerometers	Measures quasi-static and dynamic measurements. Sensor fusion using a Kalman filter improves rotational measurement.	Provides a more accurate and reliable estimate of structural rotation and deflection.	Requires significant expertise for setup, calibration, and data analysis. Limited field application, warrants further testing.	Mineral Line Railway Bridge, UK ^7^
**Vibration-based**	Tri-axial and mono-axial piezoelectric accelerometers	Operates based on the piezoelectric effect of materials to measure dynamic changes in mechanical variables (e.g., acceleration).	Offers real-time monitoring and global insights into changes in boundary conditions, such as scour.	Highly sensitive to environmental factors; requires signal filtering to reduce noise. Lack of research on a reliable scour assessment.	A71 motorway viaduct over the Loire River, Orléans, France ^82^; Kupa Karlovac Bridge, Croatia ^62^
**Vibration-based**	3-axis high-sensitivity QMEMS accelerometers	Detects scour-induced changes in natural frequencies through real-time ambient vibration measurements.	Captures alternative structural response parameters, such as spectral density and mode shapes, which reflect scour-induced behaviour changes.	Scour-induced natural frequency changes predicted by finite element models were not observed using the peak-picking method.	Baildon Bridge during scour repair program, Bradford, UK ^112^
**Vibration-based**	Broad-band high-sensitivity accelerometers with 20 Hz sampling	Monitors scour-induced changes in natural frequencies of the structure using time-frequency analysis combined with probabilistic trend detection to identify anomalies and trends.	Integrates with existing Structural Health Monitoring systems, high sensitivity to scour	Requires long-term monitoring and robust statistical processing. It may need initial calibration to filter environmental noise.	Jintang Bay Bridge, Zhoushan, China (simulation); Anqing Yangtze River Bridge, Anhui, China (field study) ^119^
**Vibration-based**	Piezoelectric acceleration sensors, finite element model	Uses ambient vibration measurements of superstructures to update finite element models for scour identification.	Incorporates mode shape variations to qualitatively detect scour. Accurately verifies findings using underwater terrain mapping.	Quantitative identification requires detailed bridge information for model updates. Effective sensor arrangements are still under investigation.	Hangzhou Bay Bridge, China ^115^
**Vibration-based**	Piezoelectric and MEMS accelerometers	Uses centrifuge modelling to scale soil properties and measure natural frequency variations caused by scour.	Natural frequency can be a potential scour indicator for integral bridges with piled foundations.	Low sensitivity of fundamental natural frequencies to scour in shallow foundations.	Laboratory test ^113^
**InSAR**	Satellite data	Analyses phase differences (interferograms) in multiple SAR images acquired over time at the same imaging geometry.	Provides millimetre-scale deformation data. Wide-area coverage. Effective under diverse conditions (e.g., night, flooding). Demonstrated potential for early warning systems.	Relies on satellite data availability and acquisition planning. Interpretation of displacement data is complex. As measurements are based on phase changes in the radar signal, deformations greater than a quarter of the radar wavelength between consecutive acquisitions is difficult to distinguish.	Tadcaster Bridge, UK ^121^; A22 Po River Bridge, Italy ^123^; Jacques Cartier and Victoria Bridges in Montreal, Canada ^122^

### 4. Summary and prospects

Selecting a robust bridge monitoring solution under flood conditions requires a comprehensive and integrated approach. Effective SHM systems should incorporate multiple monitoring techniques with cross-verification capabilities to enhance data reliability and reduce the risk of misinterpretation. Given the environmental factors that impact data accuracy, it is crucial to design SHM systems that account for these influences. For example, sonar systems work only when submerged, so pairing them with water-level monitoring sensors ensures continuous, accurate measurements. Similarly, FBG sensors used for strain measurements are sensitive to temperature changes, necessitating reference sensors to isolate strain data from temperature variations ^125– 127^.

Indirect monitoring methods, such as rotation and vibration-based monitoring and InSAR, are also subject to environmental fluctuations. To mitigate this, additional sensors that capture environmental parameters, such as temperature, wind speed and direction, humidity, and rainfall intensity, water level are essential for effective data correlation and analysis ^34, 52, 117^. Studies show that temperature fluctuations, both daily and seasonal, can correlate with changes in tilt and natural frequencies, and thus filtering and adjusting for these variations are essential for accurate structural assessments ^34, 82, 114, 118, 128^.

Currently, direct scour monitoring primarily focuses on measuring scour depth relative to allowable thresholds, often overlooking the broader structural implications of scour ^53^. Indirect methods, such as vibration-based monitoring, offer a practical means to detect a wider array of damage scenarios during flood events. Continuous indirect monitoring also generates extensive raw data, ideally suited for data-driven approaches that enable early failure detection and predictive maintenance. Typically, one year of data is needed to train machine learning models effectively, which can then automate data interpretation and enhance response times ^129^. However, field validation of vibration-based scour monitoring techniques is challenging due to the unpredictability of scour events, requiring long-term monitoring without certainty of occurrence. Consequently, research in this area is usually limited to controlled laboratory experiments and numerical simulations.

The literature review reveals a shortage of comprehensive studies integrating structural and hydraulic monitoring. This gap suggests that hybrid SHM systems, which combine direct and indirect monitoring methods with machine learning tools for real-time data analysis, represent a promising pathway toward resilient infrastructure. Table 6 summarises the key SHM technologies discussed, highlighting their strengths, weaknesses, and insights for improvement.

**Table 6.** Summary of SHM technologies for bridge monitoring in flood conditions.

SHM technology	Strengths	Weaknesses	Insights
**Photographic imagery for debris accumulation, water level, and flow velocity**	- Direct, visual monitoring - Cost-effective - Can be enhanced with AI	- Limited visibility in low-light or adverse weather conditions	AI integration for automated data analysis could enhance real-time detection accuracy and integrate multi-parameter data, such as water flow or debris density measurements.
**Load cells and pressure sensors for hydrodynamic forces**	- Direct measurement of force - Real-time data on load stress	- Sensitive to environmental conditions - Maintenance-intensive - Limited field testing	Future development could focus on the integration of direct force measurements in real-life applications.
**Fibre Bragg Grating sensors for strain and/or temperature**	- High sensitivity, long-term reliability - Immune to EM interference	- High cost - Requires specialised installation procedures and protection from flood damage	Cost reduction and multi-functional use could broaden FBG applications.
**Acoustic Doppler Current Profilers and ultrasonic profilers for flow velocity/water level**	- High precision - Multi-depth profiling	- High cost - Vulnerable to debris - Reduced performance in turbidity	Protective features to reduce debris sensitivity could extend applicability in flood conditions.
**Sonar-based systems for scour and erosion**	- Effective for detecting sub-surface scour - High-resolution riverbed data	- Limited to submerged conditions - Prone to interference from bubbles, turbidity, debris	Hybrid sonar that adapts to high and low water levels would provide year-round monitoring.
**Smart rocks and float-out devices for scour**	- Real-time sediment displacement monitoring - Cost-effective for critical events	- Float-outs provide binary data only - Risk of interference from redeposition or environmental magnetic fields	Magnetic smart rocks can provide detailed erosion patterns, but sediment redeposition needs to be managed.
**Sonar probes for direct scour monitoring**	- Precise depth measurements - Effective in direct scour measurement	- Limited coverage - Susceptible to turbulence and environmental interference	Regular maintenance and advanced filtering are needed to improve accuracy amid environmental fluctuations.
**Rotation and vibration-based indirect monitoring**	- Low-maintenance - Detects shifts in structural response	- Environmental sensitivity - Requires expertise in data interpretation	Future systems could focus on deep machine learning techniques to provide automated data-driven decision-making.
**InSAR**	Millimetre-scale deformation monitoring; wide-area coverage; works in diverse conditions	One-dimensional displacement data; complex interpretation	LOS displacement conversion into local reference systems would improve practical application and interpretation.

The integration of hybrid SHM systems that combine direct and indirect methods with AI-driven data analysis and predictive maintenance tools represents a transformative approach to bridge safety. This combination enables resilient, adaptable monitoring solutions capable of responding to increasingly severe flood scenarios. Investing in such comprehensive systems not only enhances bridge safety but also sets a foundation for smarter, future-ready infrastructure solutions.

Future research should focus on the following areas to advance SHM technologies:

- Exploring hybrid monitoring systems that combine structural and hydraulic data, offering a more comprehensive view of bridge health under stress.
- Leveraging AI and machine learning to analyse large datasets in real-time, providing automated anomaly detection and predictive maintenance alerts for faster and more efficient responses.
- Developing cost-effective sensors or modular SHM packages that allow for scalable deployment across various bridge types, making advanced monitoring accessible even for smaller or less critical structures.
- Designing adaptable SHM systems to withstand extreme weather events and address the growing impacts of climate change on infrastructure.

These directions aim to make SHM systems both technically advanced and economically feasible, ultimately supporting the development of a safer and more resilient future for bridge infrastructure.

Addressing these challenges and opportunities will require multidisciplinary collaboration among civil engineers, data scientists, hydrologists, and policymakers. Innovations in SHM must incorporate insights from diverse fields to meet the increasing demands posed by climate change and its associated risks. By fostering such collaborative efforts, SHM technologies can evolve into comprehensive, intelligent systems that not only protect critical infrastructure but also advance global resilience against environmental challenges.

## Ethics and consent

Ethical approval and consent were not required.

## Data Availability

No data are associated with this article.
