# Supplementary material for: Advancing bridge resilience: a review of monitoring technologies for flood-prone infrastructure
Source: Open Res Eur. 2025 Mar 17;5:26. Originally published 2025 Jan 27. [Version 2] doi: 10.12688/openreseurope.19232.2 (PMC12004067; doi:10.12688/openreseurope.19232.2)
Supplement: Peer review history

# Reviewer report: Reviewer response for version 2

**DOI:** 10.21956/openreseurope.21477.r52145

**Published:** 2025-04-16

**Authors:** Maryam Nasim

**Affiliations:** 1 University of Melbourne, Melbourne, Australia

The authors have fully addressed all the reviewer comments with clear, comprehensive revisions. The manuscript has been substantially improved and is now well-structured, technically sound, and ready for acceptance.

 Sincerely,

Is the review written in accessible language?

Partly

Are all factual statements correct and adequately supported by citations?

Partly

Are the conclusions drawn appropriate in the context of the current research literature?

Partly

Is the topic of the review discussed comprehensively in the context of the current literature?

Partly

Reviewer Expertise:

NA

I confirm that I have read this submission and believe that I have an appropriate level of expertise to confirm that it is of an acceptable scientific standard.

# Reviewer report: Reviewer response for version 2

**DOI:** 10.21956/openreseurope.21477.r52146

**Published:** 2025-04-14

**Authors:** Riyadh Alsultani

**Affiliations:** 1 University of Babylon, Babylon, Iraq

I appreciate the authors' efforts in addressing the reviewers’ comments and incorporating the suggested revisions. I have now reviewed the revised version of the manuscript along with their responses.

 Based on the improvements made — particularly the enhancements in the clarity of the Introduction and Summary sections, the expanded treatment of debris impact, and the valuable additions to Tables 4 and 6 — I am pleased to confirm that the revised version has satisfactorily addressed my previous concerns.

 Accordingly, I update my review status to **Approved**.

Is the review written in accessible language?

Partly

Are all factual statements correct and adequately supported by citations?

Yes

Are the conclusions drawn appropriate in the context of the current research literature?

Yes

Is the topic of the review discussed comprehensively in the context of the current literature?

Yes

Reviewer Expertise:

NA

I confirm that I have read this submission and believe that I have an appropriate level of expertise to confirm that it is of an acceptable scientific standard.

# Reviewer report: Reviewer response for version 1

**DOI:** 10.21956/openreseurope.20815.r50397

**Published:** 2025-02-20

**Authors:** Maryam Nasim

**Affiliations:** 1 University of Melbourne, Melbourne, Australia

The paper provides a comprehensive review of Structural Health Monitoring (SHM) technologies applied to flood-prone bridges, incorporating case studies and discussing various monitoring techniques. The authors have successfully highlighted the importance of SHM in mitigating bridge failures due to floods, particularly from scour, debris impact, and hydrodynamic forces. The review integrates recent advancements and proposes future research directions.

 However, there are areas that require improvements as below.

**Detail Comments:**

 1- The last paragraph in section 1 (Introduction) would be more suitable for inclusion in a summary section rather than the introduction. 

 2- In "Debris Impact" section, the effect of debris accumulation is described, but the influence of submerged objects (such as log, etc) on flow velocity has not been discussed or differentiated.

 3- While the paper provides an extensive review, there are some key SHM technologies that are briefly mentioned but not fully elaborated upon. For example, fiber optic sensors deserve more discussion.

 4- Section 3.2 ,Formula 2 introduces several notations that require clearer descriptions. Specifically, the 'shape factor' is inadequately defined. The manuscript should provide a detailed explanation of the shape factor weather its derivation or empirical basis or analytical and how its influence on the calculated outcomes. 

 5- Section 3.3 mentions float-out devices; however, the discussion lacks details on their operational mechanisms, specific applications, and suitability for different bridge types. For clarity and completeness, it is recommended to include examples of successful implementations, potential challenges, and a comparison with alternative flood resilience solutions. 

 6- One critical aspect that appears to be underrepresented is the cost analysis of the suggested methodologies. It is recommended to include a comprehensive discussion on the economic feasibility, covering implementation, maintenance, and operational costs, to improve the practical relevance and applicability of the study.

Is the review written in accessible language?

Partly

Are all factual statements correct and adequately supported by citations?

Partly

Are the conclusions drawn appropriate in the context of the current research literature?

Partly

Is the topic of the review discussed comprehensively in the context of the current literature?

Partly

Reviewer Expertise:

My primary areas of research expertise include Structural Health Monitoring of critical infrastructure and the hydrodynamic impacts of flooding, debris, and log accumulation on bridge structures. My work extensively covers vulnerability assessments of bridges under extreme flood loading, with a particular focus on failure mechanisms, debris-induced hydrodynamic forces, and structural resilience strategies. I have conducted comprehensive studies that explore the interaction between hydraulic forces and structural responses, incorporating both numerical modeling and experimental validation.Given my background, I am confident in assessing all aspects of this manuscript, especially those related to the impact of debris and flood-induced forces on bridge stability, as well as the application of SHM technologies for real-time monitoring and maintenance planning. I believe my expertise aligns well with the core topics discussed in this paper, enabling me to provide a thorough and constructive review.

I confirm that I have read this submission and believe that I have an appropriate level of expertise to confirm that it is of an acceptable scientific standard, however I have significant reservations, as outlined above.

# Response

**Published:** 2025-03-03

**Authors:** Karina Buka-Vaivade

**Affiliations:** Universita Politecnica delle Marche Dipartimento di Ingegneria Civile Edile e Architettura, Ancona, Marche, Italy

Dear Reviewer, Thank you for your valuable feedback. All recommendations have been taken into account and the article has been modified accordingly. Below there are step-by-step answers for all comments.

**Comment #1:** The last paragraph in section 1 (Introduction) would be more suitable for inclusion in a summary section rather than the introduction.

**Response:** We agree with this consideration and we removed the last paragraph of the introduction section.

**Comment #2:** In "Debris Impact" section, the effect of debris accumulation is described, but the influence of submerged objects (such as log, etc) on flow velocity has not been discussed or differentiated.

**Response:** We thank you for this valuable suggestion. We have expanded the “Debris Impacts” section to clearly differentiate the effects of floating and submerged debris, and added relevant references. Additionally, we have incorporated findings from experimental studies that demonstrate how submerged debris influences local scour based on its position relative to the flow surface and alters flow dynamics.

**Comment #3:** While the paper provides an extensive review, there are some key SHM technologies that are briefly mentioned but not fully elaborated upon. For example, fiber optic sensors deserve more discussion.

**Response:** We have reviewed our discussion on fiber optic sensors and made several enhancements. We added cross-referencing between Section 3.2 and Table 4 where fiber optic sensing is discussed. We added clarification to Distributed Fiber Optic Sensors (DFOS) in comparison to Fiber Bragg Gratings (FBGs). As well, we added additional example of a Fiber-Optic Distributed Temperature Sensing (DTS) device in Table 4.

**Comment #4:** Section 3.2, Formula 2 introduces several notations that require clearer descriptions. Specifically, the 'shape factor' is inadequately defined. The manuscript should provide a detailed explanation of the shape factor weather its derivation or empirical basis or analytical and how its influence on the calculated outcomes.

**Response:** We apologize for missing important details in that discussion. We have revised Section 3.2 to provide a clearer definition of the shape factor (k), explaining its empirical basis, how it is derived, and its influence on the calculated hydrodynamic forces. The updated text clarifies that the shape factor accounts for the impact of cross-sectional geometry on drag, with values taken from Eurocode (EN 1991-1-6, 2005). Additionally, we have included a brief discussion on how different shapes influence flow separation and turbulence, which directly affects force estimation.

**Comment #5:** Section 3.3 mentions float-out devices; however, the discussion lacks details on their operational mechanisms, specific applications, and suitability for different bridge types. For clarity and completeness, it is recommended to include examples of successful implementations, potential challenges, and a comparison with alternative flood resilience solutions.

**Response:** We have expanded Section “ **iii. Embedded sensors and smart rocks**” to provide more details on float-out devices. Specifically, we have clarified their operational mechanism (tethered vs. untethered, transmitter alerts); included an example of field deployment and scour detection (SR 101 bridge, California); and highlighted their advantages, limitations and the need of combining them with other technologies for comprehensive scour assessment.

**Comment #6:** One critical aspect that appears to be underrepresented is the cost analysis of the suggested methodologies. It is recommended to include a comprehensive discussion on the economic feasibility, covering implementation, maintenance, and operational costs, to improve the practical relevance and applicability of the study.

**Response:** Following your suggestion, we have expanded the discussion on economic feasibility. Specifically, Table 6 now provides a comparative overview of SHM technologies, addressing cost considerations. However, we believe that providing a very detailed discussion about economic aspects is outside the scope of the article, as well as difficult to do because instrumentation, installation, and management of the monitoring system have different costs in different parts of the world, also related to the different sensitivities of infrastructure management operators.

# Reviewer report: Reviewer response for version 1

**DOI:** 10.21956/openreseurope.20815.r50394

**Published:** 2025-02-18

**Authors:** Riyadh Alsultani

**Affiliations:** 1 University of Babylon, Babylon, Iraq

- The introduction is well-structured, clearly stating the background and significance of the study. Some sentences are lengthy and could be simplified for better readability.

 - The conclusion section is clear and well-organized, summarizing the main findings of the study. However, it can be made more concise by avoiding repetitive phrases and ensuring that conclusions are directly tied to the study’s objectives.

Is the review written in accessible language?

Partly

Are all factual statements correct and adequately supported by citations?

Yes

Are the conclusions drawn appropriate in the context of the current research literature?

Yes

Is the topic of the review discussed comprehensively in the context of the current literature?

Yes

Reviewer Expertise:

Hydraulic structures

I confirm that I have read this submission and believe that I have an appropriate level of expertise to confirm that it is of an acceptable scientific standard, however I have significant reservations, as outlined above.

# Response

**Published:** 2025-03-03

**Authors:** Karina Buka-Vaivade

**Affiliations:** Universita Politecnica delle Marche Dipartimento di Ingegneria Civile Edile e Architettura, Ancona, Marche, Italy

Dear Reviewer, We appreciate your insightful feedback. All your recommendations have been considered, and the article has been revised accordingly. Below, you will find detailed responses to each of your comments.

**Comment #1: **Some sentences in introduction are lengthy and could be simplified for better readability.

**Response:** Lengthy sentences in introduction part have been simplified for better readability.

**Comment #2: **The conclusion section can be made more concise by avoiding repetitive phrases and ensuring that conclusions are directly tied to the study’s objectives.

**Response:** We thank you of this suggestion and we have revised the summary section to enhance conciseness, removing redundant phrasing and ensuring that findings are clearly tied to the study’s objectives. Additionally, Table 6, which contains a comparative overview of SHM technologies for bridge monitoring in flood conditions, has been refined for clarity.
